# Antimicrobial Essential Oil Formulation: Chitosan Coated Nanoemulsions for Nose to Brain Delivery

**DOI:** 10.3390/pharmaceutics12070678

**Published:** 2020-07-17

**Authors:** Federica Rinaldi, Alessandra Oliva, Manuela Sabatino, Anna Imbriano, Patrizia N. Hanieh, Stefania Garzoli, Claudio M. Mastroianni, Massimiliano De Angelis, Maria Claudia Miele, Marcela Arnaut, Federica Di Timoteo, Carlotta Marianecci, Rino Ragno, Maria Carafa

**Affiliations:** 1 Department of Drug Chemistry and Technology, Sapienza University of Rome, 00185 Rome, Italy; federica.rinaldi@uniroma1.it (F.R.); anna.imbriano@uniroma1.it (A.I.); patrizianadia.hanieh@uniroma1.it (P.N.H.); stefania.garzoli@uniroma1.it (S.G.); maria.carafa@uniroma1.it (M.C.); 2 Department of Public Health and Infectious Diseases, Sapienza University of Rome, 00185 Rome, Italy; alessandra.oliva@uniroma1.it (A.O.); claudio.mastroianni@uniroma1.it (C.M.M.); massimiliano.deangelis@uniromal.it (M.D.A.); mariaclaudia.miele@uniroma1.it (M.C.M.); marcela.arnaut@uniroma1.it (M.A.); federica.ditimoteo@uniroma1.it (F.D.T.); 3 Department of Drug Chemistry and Technology, Rome Center for Molecular Design, 00185 Rome, Italy; manuela.sabatino@uniroma1.it

**Keywords:** *Thymus vulgaris*, *Syzygium aromaticum*, essential oils, nanoemulsions, chitosan coating, antibacterial activity, nose to brain delivery

## Abstract

Brain infections as meningitis and encephalitis are attracting a great interest. Challenges in the treatment of these diseases are mainly represented by the blood brain barrier (BBB) that impairs the efficient delivery of even very potent drugs to reach the brain. The nose to the brain administration route, is a non-invasive alternative for a quick onset of action, and enables the transport of numerous medicinal agents straight to the brain thus workarounding the BBB through the highly vascularized olfactory region. In this report, *Thymus vulgaris* and *Syzygium aromaticum* essential oils (EOs) were selected to be included in chitosan coated nanoemulsions (NEs). The EOs were firstly analyzed to determine their chemical composition, then used to prepare NEs, that were deeply characterized in order to evaluate their use in intranasal administration. An in vitro evaluation against a collection of clinical isolated bacterial strains was carried out for both free and nanoemulsioned EOs. Chitosan coated NEs showed to be a potential and effective intranasal formulation against multi-drug resistant Gram-negative bacteria such as methicillin-susceptible *Staphylococcus aureus* and multi-drug resistant Gram-negative microorganisms including carbapenem-resistant *Acinetobacter baumannii* and *Klebsiella pneumoniae*.

## 1. Introduction

The ever increasing interest in nose to brain delivery is demonstrated by a huge scientific document production as reported by a simple search in Scopus. During the last decade, around 4060 documents have been published on the nose to brain topic, a two-fold increase compared to papers published in the previous decade (1999–2009) and the same trend is for papers on microemulsions and intranasal administration (133 documents 1997–2020 but with an evident increase in published papers since 2007) and for nanoemulsions and intranasal administration (164 documents from 1997–2020 but with an evident increase in published papers since 2009), the situation is quite different coupling intranasal administration with essential oils, only 33 documents, in the same time interval are present. The intranasal (IN) route of administration is a challenge to conventional drug delivery approaches for effective and efficient delivery of drugs to the brain and central nervous system (CNS). The clinical potency of any therapeutic agent is not only dependent on its bioavailability but its ability to penetrate the protective layer, i.e., BBB and cerebrospinal fluid (CSF), which represent the major obstacles for treatment of nervous system diseases. Therefore, the choice of the way of administration and the optimization of the formulation for brain targeting are fundamental to design an innovative and promising therapeutic strategy.

CNS diseases including Alzheimer’s disease and Parkinson’s disease, strokes, and brain cancers are the world’s leading causes of disability, and thus have been attracting more and more attentions. Nevertheless, a great focus is given to brain infections and, in particular, on the management of CNS infections caused by difficult-to-treat microorganisms, such as multi-drug resistant bacteria [1]. Even in these diseases, the failure of brain infection treatment is mostly attributed not to the potency of the drugs, but to the BBB that impairs the efficient delivery of the drugs to the brain. Another major therapeutic challenge is represented by agents usually causing nosocomial CNS infections (i.e., post neurosurgical meningitis with or without brain involvement; infections of external ventricular drainage system; infections of ventriculoperitoneal shunt), which often show a multi-drug resistant phenotype [2,3].

In the majority of cases, these agents are methicillin-resistant *Staphylococcus aureus* (MRSA) and MDR Gram-negative bacteria such as carbapenem-resistant *Klebsiella pneumoniae* (CR-Kp), carbapenem-resistant *Acinetobacter baumannii* (CR-Ab), and carbapenem-resistant *Pseudomonas aeruginosa* (CR-Pa) [4]. When a nosocomial central nervous system infection is caused by the abovementioned microorganisms, physicians deal with the problem of the multi-drug resistance which limits the availability of effective drugs and accounts for the high mortality observed in these infections [5,6]. In fact, in such extreme cases, in addition to systemic administration of the drugs, the therapeutic approach is also based on the intrathecal route, which, obviously, carries a not negligible rate of adverse events [4,7]. Therefore, in consideration of the therapeutic challenge represented by these types of infections, finding an alternative and effective way of antimicrobial administration (i.e., the nose-to-brain), which might be able to replace the intrathecal administration with a lower toxicity profile, might represent a significant advance in the management of CNS infections caused by MDR bacteria [8]. Therefore, there is a growing need to develop new agents to treat bacterial infections such as highly active new antibiotics with diversified mechanisms of action or developing nanocarriers with a high targeting efficiency or exploring a more efficient administration route, able to circumvent BBB providing a direct and rapid delivery into the brain [7,9].

The nose to the brain (N2B) administration route is a non-invasive alternative for a quick onset of action, able to transport numerous medicinal agents with various molecular weights straight to the brain, avoiding the BBB through the highly vascularized olfactory region, thus decreasing the whole cost of therapy, improve patient’s compliance, and blunt drug’s systemic side effects [8].

The IN delivery can occur through three possible pathways: by the systemic circulation, by the olfactory nerve, and by the trigeminal, nevertheless the detailed mechanisms involved in N2B are yet not clear [10]. However, the potential advantages of nasal administration are intriguing, there are some limitations, which enable challenges to develop new delivery strategies. Among the major limitation is nasal-mucociliary clearance [11], a normal physiological phenomenon that protects the lungs from inhaled harmful agents, such as pathogens or microorganisms. Enzymatic degradation at the nasal mucosa [12] is another limitation especially for protein and peptide agents, that can be degraded by enzymes present in the lumen while crossing the epithelial barrier reducing bioavailability. Other limitations include nasal toxicity due to some therapeutic agents and absorption enhancers, problem to cross nasal mucosa for high molecular weight drugs, chances of severe damage of nasal cavity by repeated drug administration [13].

Considering the above limitations scenario, nanoemulsions (NEs) might represent a promising platform for therapeutic agent delivery into the brain by IN delivery [14]. NEs are nano-sized globules (of size ranging from 10 to 100 nm) composed by the oil/water biphasic liquid system stabilized by means of a combination of surfactant-co surfactant, they are translucent systems, kinetically stable [15,16,17]. NEs are able to incorporate lipophilic drugs by solubilizing them in the lipid phase, that itself can possess therapeutic activities able to act in a synergic manner with the loaded drug [13].

Surfactants are the NEs integral components that on one side stabilize NEs and affect the NEs particle size though having a significant effect on nasomucosal permeation and on intranasal permeation enhancement by altering the fluidity of the membrane [18].

The choice of oil and surfactant pair is of crucial importance for the design and preparation of the desired nanoemulsion and at the same time for preparing functional NEs in which oil properties can have a synergic effect with the entrapped drug in order to enhance the activity of the overall system, and in which the surfactant concentration should be kept as low as possible to balance between drug permeation and no or less toxic effect [19].

Compared to other drug delivery systems, numerous studies indicate that mucoadhesive NEs are more effective for the direct N2B delivery due to their enhanced retention of the system on the nasal mucosa [13]. Mucoadhesiveness is obtained by including mucoadhesive materials such as chitosan or its derivatives that establish interactions with the mucose and increase nasal cavity retention [20,21].

Chitosan, a biodegradable natural polymer, was reported to enhance both penetration and drug absorption through nasal mucosa and to delay mucociliary clearance. Chitosan is also well tolerated and has an excellent biocompatibility [22]. Several studies confirm a chitosan double role, demonstrating that chitosan-coated NEs had the highest flux and permeation across the nasal mucosa compared to uncoated NEs [23].

Among the new antimicrobial remedies, EOs have received a great amount of attention particularly due to the lack of induced resistance [24], their potentiality as antimicrobial agents [25,26], and considering the recent reports on their efficacy in the EO loaded nanosystem [27,28] from an antimicrobial screening of an in house library of EOs, *Thymus vulgaris* (also termed thyme, TV) and *Syzygium aromaticum* (also named *Eugenia caryophyllata* or cloves, SA). The derived EOs were selected for their wide activity spectra against a panel of Gram-positive and Gram-negative microorganisms (i.e., *Staphylococcus aureus*, *Klebsiella pneumoniae*, *Acinetobacter baumannii*) to prepare different NEs as potential IN formulations and evaluate their antimicrobial efficacy.

In particular, the aim of the study was the preparation of TV and SA essential oils (TVEO and SAEO) chitosan NEs and their in vitro evaluation against a collection of bacterial strains of clinical concern including methicillin-susceptible *Staphylococcus aureus* (MSSA), MRSA, *Escherichia coli*, MDR Gram-negative bacteria (CR-Kp, CR-Ab, and CR-Pa). The selected surfactant, sorbitane monolaurate (Span 20) is listed as acceptable surfactants/co-surfactants for nasal formulations in the US FDA Inactive Ingredients Database (IID, January 2019). In addition, for comparison purposes the in vitro antibacterial effectiveness of TVEO and SAEO against the abovementioned microorganisms was also evaluated.

## 2. Materials and Methods

### 2.1. Materials

Span 20, Hepes salt {*N*-(2-hydroxyethyl) piperazine-*N*-(2-ethanesulphonic acid)}, pyrene, 1,6-diphenyl-1,3,5-hexatriene (DPH), mucin from porcine stomach type II powder, thymol, eugenol, vancomicin, and meropenem were purchased from Sigma-Aldrich (Milan, Italy). All other products and reagents were of analytical grade. TVEO and SAEO were a generous gift of Farmacia Crimi of Rocco Crimi (Rome, Italy).

### 2.2. EOs Chemical Analysis

The gas chromatographic/mass spectrometric (GC/MS) analysis was carried out with a GC-MS and GC-FID using a Turbomass Clarus 500 GC-MS/GC-FID from Perkin Elmer instruments (Waltham, MA, USA). A Stabilwax fused-silica capillary column (Restek, Bellefonte, PA, USA) (60 m × 0.25 mm, 0.25 μm film thickness) was used with helium as the carrier gas (1.0 mL/min). The GC oven temperature was kept at 60 °C for 5 min and programmed to 220 °C at a rate of 5 °C/min, and kept constant at 220 °C for 30 min. MS was taken at 70 eV. The mass range was from 30 to 350 *m/z*. 1 μL of oil was diluted in 1 mL of methanol and 1 μL of the solution was injected into the GC injector at the temperature of 280 °C [25,29].

The identification of the constituents was made by comparison of the obtained mass spectra for each compound with those reported in mass spectra NIST, NBS, and Wiley Libraries. Linear retention indices (LRIs) were calculated using a mixture of aliphatic hydrocarbons (C8-C30, Ultrasci), injected directly into the GC injector at the same conditions reported above. Relative percentages for quantification of the separated components were calculated by an electronic integration of the GC-FID peak areas using the normalization method without the use of correction factors. All analyses were repeated twice.

### 2.3. Nanoemulsion Preparation

NEs were prepared using Span 20 and two different essential oils, TVEO and SAEO (Table 1) at the same weight ratios [30,31]. The oil/surfactant ratio that has been used is the result of a screening test of more values and this ratio was selected to obtain a NE size around 100 nm.

Briefly, a mixture composed by either TVEO or SAEO, Span 20 and Hepes buffer (10^−2^ M, pH 7.4) were vortexed for about 5 min allowing the emulsion formation. The surfactant concentration in the samples was always remarkably above CMC (CMC in water at 20 °C: Tween 20: 0.059 mM; CMC for Span 20: Not detectable). Each emulsion with microscale droplets was sonicated for 5 min at 60 °C using a tapered microtip operating at 20 kHz at an amplitude of 18% (Vibracell-VCX 400, Sonics, Taunton, MA, USA) to obtain NEs. The chitosan solution was prepared by dissolving chitosan low molecular weight in an acetate buffer (0.2 M, pH 4.4) up to a final concentration of 3 mg/mL. The obtained solution was stirred overnight.

Chitosan coating of NEs was obtained by adding the chitosan solution, opportunely diluted in distilled water, to the different samples with a 1:1 volumetric ratio [21]. The obtained suspension was stirred for 3 h at room temperature to achieve chitosan-coated NEs (C-SA-NEs and C-TV-NEs).

When necessary, the fluorescent probes pyrene (4 mM), Nile Red (0.32 mg·mL^−1^), and DPH (2 × 10^−4^ M) were added to the surfactant/oil mixture in the first step of the NEs preparation method.

### 2.4. Dynamic Light Scattering Measurements

Droplet size distribution and the ζ-potential of the NEs were measured at the temperature of 25 °C by dynamic light scattering (DLS), using a Zetasizer Nano ZS90 (Malvern Instruments Ltd., Worcestershire, UK), equipped with a 5 mW HeNe laser (wavelength λ = 632.8 nm) and a digital logarithmic correlator. The normalized intensity autocorrelation functions were detected at a 90° angle and analyzed by using the Contin algorithm in order to obtain the decay time of the electric field autocorrelation functions. Here, the correlation function is fitted against longer periods of time and provides monomodal size distribution peaks. The PDI for DLS typically depicts the intensity of light scattered by various fractions of the particles differing in their sizes and is calculated by (width/mean) two for each peak. While the PDI of ≤0.1 is considered to be highly monodisperse values of 0.1–0.4 and >0.4 are considered to be moderately and highly polydisperse, respectively [32].

### 2.5. NEs Stability Evaluation

To assess colloidal stability at different temperatures, the NEs formulations were stored at 4 and 25 °C for a period of 90 days. Samples from each batch were withdrawn at definite time intervals (1, 30, 60, and 90 days) and the ζ-potential, the mean of hydrodynamic diameter, and PDI were determined as previously described.

Biological studies were also carried out in the presence of artificial simulated cerebrospinal fluid (aCSF) (pH 7.3) to evaluate the stability of vesicular systems after intranasal administration. aCSF was prepared according to McNay et al. [33]. Mixtures of NEs and 45% aCSF were prepared and incubated at 37 °C. Analyses were performed at different time intervals (0, 0.5, 1, 2, and 3 h) by DLS (Nano ZS90, Malvern, UK) to evaluate variations of particle size, ζ-potential, and polydispersity index.

### 2.6. Fluorometric Measurements

By fluorescent techniques the oil drop features (microviscosity and polarity) were investigated. Pyrene loaded NEs were prepared by adding Pyrene (4 mM) to NEs components (same preparation method described above). The lateral distribution and the mobility of the probe inside the oil phase were studied by fluorescence measurements. Pyrene is a fluorescent probe and its monomer exhibited a spectrum characterized by five emission peaks (I_1_-I_5_) while its excimer shows only one peak (I_E_). Pyrene, at an opportune concentration, can form the intramolecular excimer based on the viscosity of the probe microenvironment. The fluorescence signals emitted by Pyrene loaded NEs were scanned (λ = 350–550 nm) using the luminescence spectrometer (LS5013, PerkinElmer, Waltham, MA, USA). This ratio increases as the polarity of the medium rises. Fluorescence data can be referred to the probe mobility in the oil phase together with the hydrophobic portion of the surfactant. The presence of pyrene intramolecular excimers depends on the rate of conformational change of the molecule, which is sensitive to the viscosity of the probe microenvironment [34].

The DPH solution (2 × 10^−4^ M) was previously evaporated to obtain the dry powder to be mixed with EOs. The mixture was vortexed and successively added to the Span 20 surfactant (Table 1), 5 mL of Hepes buffer, and the resulting microemulsion mixed with vortex was sonicated at 60 °C and 18% amplitude for a period of 5 min. Fluorescent measurements were performed (λ = 350–425 nm) using a luminescence spectrometer (LS5013, PerkinElmer). The fluorescence anisotropy (r) was determined by means of Equation (1) [30,31,32].

(1) $$\mathrm{Anisotropy}\;(r)=\frac{I_{\mathrm{VV}}\;-\;(I_{\mathrm{VH}}\;\times \;G)}{I_{\mathrm{VV}}\;+\;\left(2I_{\mathrm{VH}}\;\times \;G\right)}$$

where I_VV_, I_VH_, I_HV_, and I_HH_ are fluorescent intensities, and subscript V (vertical) and H (horizontal) represent the orientation of polarized light. G factor is the ratio of sensitivity of the detection system for vertically and horizontally polarized light.

### 2.7. Preparation of Mucin Solution, Mucin-NEs Interaction Studies, and pH Measurements

Mucin powder was dissolved in the Hepes buffer (2 mg/mL, pH = 6) and the resulting solution was stirred overnight at 34 °C [35]. Specific parameters, including temperature (30 °C), concentration of mucin (2 mg/mL), and pH value (6.3–6.7), were controlled in the mucin interaction study to mimic the conditions in the nasal mucosal site. The mucin solution (2 mg/mL) was mixed with 1:1 suspension of coated and uncoated chitosan NEs, and incubated at 30 °C [30,34]. The particle size and ζ-potential were measured by DLS (Nano ZS90, Malvern, UK) at 0, 5, 10, and 15 min, to determine the time needed for the chitosan coated and uncoated NEs–mucin complex formation and the stability of the complex. Data collected showed the system to be stabilized after 15 min. The interaction between the chitosan coated and uncoated NEs and mucin was also evaluated by performing fluorescence turbidity measurements using a luminescence spectrometer (LS5013, PerkinElmer) at Ex/Em 600/600 nm [36].

The mucoadhesive strength was determined by the method proposed by Sandri et al. [37]. The percent binding efficiency of chitosan coated and uncoated NEs to mucin was determined by mixing 1 mL of porcine mucin suspension (2 mg/mL in the Hepes buffer) with the same volume of C-TV-NEs and C-SA-NEs or TV-NEs and SA-NEs. The mucin alone and the mixtures with chitosan coated and uncoated NEs were then centrifuged at 18,000 rpm for 30 min at a temperature of 4 °C. The concentration of free mucin in the supernatant was determined at 255 nm using an UV spectrophotometer (Perkin-Elmer lambda 25 UV/Vis). The mucin binding efficiency (MBE) of C-TV- and C-SA-NEs or TV-NEs and SA-NEs was calculated from the following equation:

(2) $$\mathrm{MBE}\;\left(\%\right)=\frac{\mathrm{Total}\;\mathrm{mucin}-\mathrm{Free}\;\mathrm{mucin}}{\mathrm{Total}\;\mathrm{mucin}}\;\times \;100$$

To determine the pH values of formulations the pH-meter was employed (pH-meter Hanna instruments HI2211). The pH electrode and the temperature sensor were immersed into NEs suspension. The suspension was stirred with a magnetic bar (~30 s) with the same stirring rate as for calibration to obtain the best results. The measure was carried out three times for each sample at the temperature of 20 °C.

### 2.8. In Vitro Release Study

NEs were prepared with Nile Red (NR) a lipophilic probe at the concentration of 0.32 mg/mL. The experiments were carried out using dialysis tubes (molecular weight cut-off 8000 and 5.5 cm^2^ diffusing area) and following the NR increase in an external medium at definite time intervals, for 24 h at 37 °C. The release medium was gently magnetically stirred during the experiment. Sample volumes (1 mL) were withdrawn from the solution to perform UV analyses and then reinserted back in the external medium. The released NR was detected by means of a spectrophotometer (Perkin-Elmer, Lambda 3a, UV-VIS spectrometer, Waltham, MA, USA) and the analyses were carried out evaluating the NR absorbance at 559 nm. Aliquots were analyzed immediately after sampling. All release experiments were carried out in triplicate. For the reason described in Section 2.4, only NR release from the uncoated NEs was determined and only uncoated NEs were tested for antimicrobial activity and time-kill studies.

### 2.9. Diluted Essential Oils and NEs

The TVEO and SAEO and their NEs antibacterial activity was expressed as % *v/v*. TVEO and SAEO were initially dissolved in dimethylsulphoxide (DMSO 50%) or water, to obtain a complete solubilization whereas NEs were dispersed in Hepes. The used DMSO concentration did not interfere with bacterial viability.

### 2.10. Bacterial Strains

For the antimicrobial activity determination, the following microorganisms were used: *Staphylococcus aureus* subsp. aureus-MSSA (ATCC 29213), *Escherichia coli* (ATCC 25922), methicillin-resistant *Staphylococcus aureus* (MRSA clinical strain isolated from skin), carbapenem-resistant *Klebsiella pneumoniae* (CR-Kp clinical isolated strain isolated from urine), carbapenem-resistant *Acinetobacter baumanni* (CR-Ab clinical strain isolated from sputum), and carbapenem-resistant *Pseudomonas aeruginosa* (CR-Pa clinical strain isolated from bronchoalveolar lavage). After bacterial storage on the cryovial bead preservation system (Microbank; Pro-Lab Diagnostics, Richmond Hill, ON, Canada) at −80 °C, the inoculum was prepared by spreading one cryovial bead on a blood agar plate and incubated overnight at 37 °C. One colony was re-suspended in 5 mL tryptic soy broth (TSB) and incubated at 37 °C without shaking. Overnight cultures were then adjusted to a turbidity of 0.5 McFarland, corresponding to ≈1 × 108 CFU/mL.

### 2.11. Antimicrobial Activity of EO and NEs

EOs minimal inhibitory concentration (MIC) and minimal bactericidal concentration (MBC) were determined by the macro dilution broth method [38]. Briefly, two-fold serial dilutions of each EO and their NEs were prepared in a 2 mL Mueller Hinton broth (MHB) in borosilicate glass tubes and incubated for 24 h at 37 °C. MIC was defined as the lowest concentration of EO that completely inhibited visible growth whereas the bactericidal activity was defined as ≥3-log10 CFU/mL reduction of the initial bacterial count after 24 h of incubation. The used final bacterial inoculum was ~5 × 10^5^ CFU/mL. The antimicrobial activity of both EOs and their NEs was expressed as % *v/v*.

### 2.12. Disk Diffusion (DD) Assay

The antimicrobial activity of the tested EOs was compared against the same selection of antibiotic—resistant and—sensitive bacterial strains described above by using DD assays.

For the DD analysis, 10 μL of absolute (100% *v/v*) EO were inoculated onto individual 6-mm filter paper discs and placed on Mueller Hinton Agar (MHA) plates containing ~1.5 × 10^8^ CFU/mL of the tested microorganism. The antimicrobial effect was assessed by measuring the inhibition zone (expressed as mm) after 24 h of incubation at 37 °C.

### 2.13. Time-Kill Studies of NEs

Given the observed high antibacterial effect of SA-NEs and TV-NEs against CR-Ab and CR-Kp, their activity was further evaluated by time-kill studies performed in the logarithmic growth phase using an initial bacterial inoculum of ~5 × 10^5^ CFU/mL.

Killing curves were performed in boro-silicate glass tubes in a final volume of 10 mL MHB which were further incubated at 37 °C. At 2, 4, 6, 8, and 24 h time points, 1 mL aliquots were sampled and washed with a 0.9% saline solution in order to prevent the carry-over effect. Ten-fold dilutions were then plated on a Muller-Hinton agar and the number of CFUs was determined. The medium without NEs was used as the growth control. Bactericidal activity was defined as ≥99.9% (i.e., ≥3 log10 CFU/mL)) reduction of the initial bacterial count at each time point. With respect to the MIC values obtained as described above, the following concentrations of NEs were used: 0.5 × MIC (0.015% *v/v*), 1 × MIC (0.03% *v/v*), 2 × MIC (0.06% *v/v*), 4 × MIC (0.125% *v/v*).

### 2.14. Statistical Analysis

Results are expressed as the mean of three independent experiments ± standard deviation. The data were statistically analysed using Minitab-18 and Excel for Mac 2011. Significance of data was performed using one-way analysis of variance (ANOVA), and differences were significantly different when *p* < 0.05.

## 3. Results

### 3.1. EOs Chemical Analysis

The chemical analysis of TVEO showed the presence of 21 chemical components (Table 2) among which thymol was the most abundant (44.4%) followed by o-cymene (18.2%) and linalool (7.5%). SAEO was characterized by five components and eugenol was the major constituent (80.1%). Eugenol acetate (9.9%) and β-caryophyllene (8.7%) were also present.

The GC/MS analysis was successfully used to evidence the different chemical composition of the two analyzed EOs.

### 3.2. Dynamic Light Scattering Measurements

As for formulation attributes, the nanoemulsion particle size (determined by dynamic light scattering), zeta potential, and PDI need to be measured.

By means of DLS, the size expressed as hydrodynamic diameter was measured and SA- and TV-NEs show a mean size of around 100 nm (Table 3). As reported by Ahmad et al. [39], nanoemulsions with a 100 nm average droplet size was transported to the olfactory bulb via olfactory and trigeminal nerve at a higher rate and extent, compared to the nanoemulsion with 900 nm average size [13]. All these formulations showed a PDI of 0.2 and, as previously described, this polydispersity index is important in drug pharmacokinetics, as lower values indicate an enhanced probability of a more uniform absorption through the nasal mucosa and higher values may lead to pharmacokinetic irregularity and variability in the therapeutic outcome [40].

The ζ-potential evaluation is of fundamental importance to investigate particle interactions with biological milieu and to predict stability over time. It is recommended that its absolute value should be above 30 mV for maximum stability [13]. The obtained average ζ-potential values for SA- and TV-NEs were negative enough to assure good time stability, in fact are more negative than −30 mV and are around −40 mV.

Upon the chitosan coating, both samples C-SA- and C-TV-NEs showed a charge inversion and the ζ-potential turned to positive values confirming the coating and to enable the interaction with the negatively charged nasal mucosal membrane likely due to the establishment of electrostatic interactions with the sialic acid residues (Table 3). Although the chitosan coating can induce an increase of droplet size the PDI values remained in the nm range of a monodisperse preparation (Table 3).

### 3.3. Fluorimetric Measurements

Qualitative and quantitative effects of different oils on features of the dispersed oily phase, were investigated by pyrene experiments (Table 4). Polarity values, described by the ratio of I_1_/I_3_, were similar for all samples ranging from 0.79 to 0.92, thus indicating that the presence of the two different oils, but of the same surfactant (Span 20) had no effect on the oil phase microviscosity and polarity [34]. Fluidity measurements showed, for both samples, a quite fluid oil phase stabilized by the surfactant layer likely due to the presence of Span 20 (HLB = 8.6 at 25 °C in water) that adopt a rigid structure around the oil drop while maintaining a dynamic fluid state in the droplet inner core. The presence of the chitosan coating did not vary the parameters related to the oily phase, thus demonstrating that the coating was a superficial phenomenon that did not influence the inner structure of the nanoemulsion.

### 3.4. NEs Stability Evaluation

**Figure 1:**
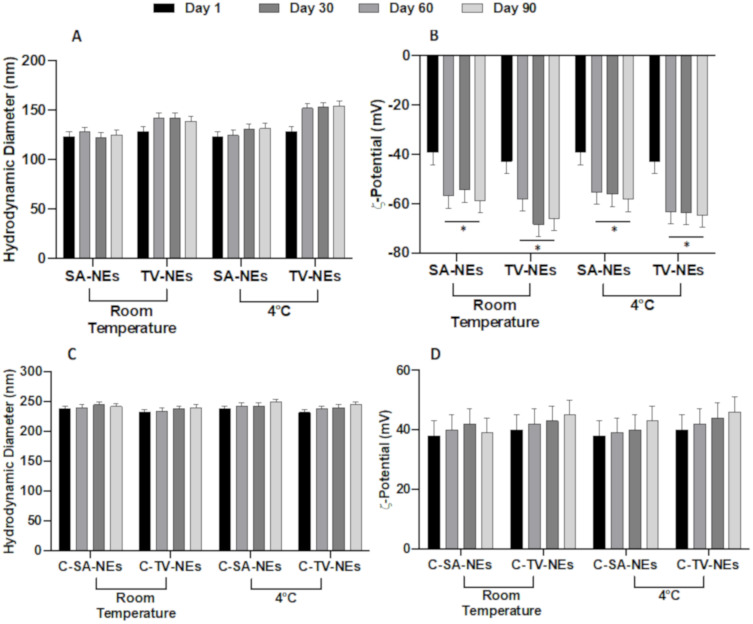
Physical stability studies in terms of hydrodynamic diameter and ζ-potential variations of uncoated (SA- and TV-NEs) (**A**,**B**) and of chitosan coated nanoemulsions (C-SA- and C-TV-NEs (**C**,**D**) up to 90 days at two different storage temperatures (* = *p* < 0.05 when compared with day 1).

As above reported, SA- and TV-NEs ζ-potential is quite negative to assure a good time stability, as demonstrated by stability studies, carried out at 4 and 25 °C for a period of 90 days. For both NE formulations, no significant variations in size at the two considered temperatures were observed throughout the analyzed period (Figure 1A,B). A significative variation in zeta potential values occurred for both samples after 1 week. ζ-potential became more negative assuring a good stability avoiding nanoemulsion droplet coalescence. The similar stability could be not only the effect but also the consequence of similar chemical-physical features. After the chitosan coating, the stability of the formulation (Figure 1C,D), in terms of size and ζ-potential variations, was maintained, probably due to the high positive charge of the droplet surface.

To date, no concrete evidence confirms N2B transport of intact nanocarriers that could be considered beneficial since excipient accumulation in the brain might cause undesirable adverse side effects. Probably, the chitosan on the NEs surface should be retained on the olfactory epithelium, and the nanoemulsion or the drug alone should be able to cross the nasal mucosa. For this reason, only the uncoated NEs stability in aCSF was determined.

**Figure 2:**
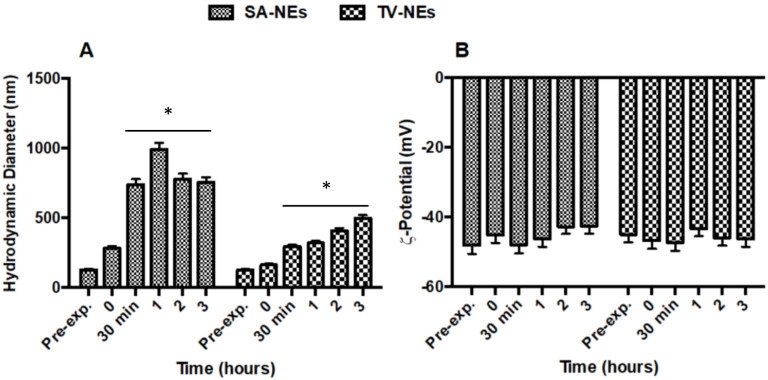
Variations of hydrodynamic diameter (**A**) and ζ-potential (**B**) of SA- and TV-NEs in the presence of aCSF up to 3 h (* = *p* < 0.05 when compared with *t* = 0).

To assess biological stability, hydrodynamic diameter and ζ-potential variations were evaluated in an aCSF medium. No statistically significant changes were observed for TV-NEs either in size within 3 h after addition of aCSF or ζ-potential for (Figure 2A,B). On the other hand, SA-NEs hydrodynamic diameter appeared to be affected by the presence of aCSF, without any ζ-potential variation.

### 3.5. Mucoadhesive Studies

The interaction between mucin (the main component in mucus) and coated and uncoated SA- and TV-NEs was evaluated determining the differences in size and surface charge before and after the addition of mucin. The pH value was evaluated to confirm for all formulations a suitable pH for nasal administration (3.5 < pH < 6.4) [41].

The hydrodynamic diameter, ζ-potential, pH, and turbidity values are listed in Table 5 and they are all compatible with the same mucin interaction scheme for both coated and uncoated SA- and TV-NEs. Surprisingly, from the analyses of the above parameters of the uncoated NEs in contact with the mucin, an increase in the size and a reduction of the ζ-potential can be seen; thus demonstrating a variation in the surface characteristics of the NEs. The value of the pH remained constant and compatible with nasal administration. Turbidity values, after addition of mucin, assume intermediate values between nanoemulsions and mucin alone, confirming the interaction of the two dispersions [42].

The hydrodynamic diameter, ζ-potential, pH, and turbidity values obtained after mucin addiction to chitosan coated nanoemulsions were monitored (Table 6). The more relevant data was the ζ-potential value inversions after chitosan coated nanoemulsion interactions with the mucin. It has been extensively reported [39,43] that the positively charged chitosan on nanoemulsion surface interacts with the anionic sites present in the mucous layers, mainly sialic acid, affecting the permeability of the epithelial membrane by the transient opening of the tight junctions in the epithelial cells of the mucosal barriers.

Moreover, in this case the pH remained constant and compatible with nasal administration and turbidity values, after the addition of mucin, assuming intermediate values between nanoemulsions and mucin alone, confirming the interaction of the two dispersions. The PDI values remained constant as reported in Table 5 and Table 6.

**Figure 3:**
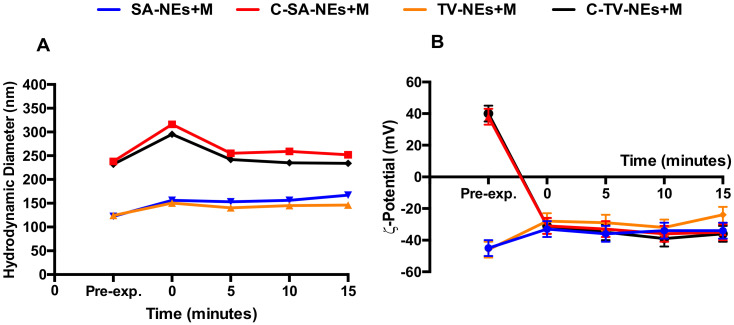
Kinetic interaction between coated and uncoated NEs and mucin, evaluated by hydrodynamic diameter (**A**) and ζ-potential (**B**) variations by DLS measurements. Panel A: Error bars are smaller than the symbol size.

The variation over time of hydrodynamic diameter and ζ-potentials were also analyzed (Figure 3A,B). It was possible to notice that after mucin addiction, the dimensions showed a slight increase and for uncoated samples the ζ-potential remained unchanged, on the contrary the ζ-potential of chitosan coated NEs became negative. These changes were immediate and the obtained complexes remained stable during the time interval observing a very important issue as it could be used to predict the in vivo sample stability [42].

**Figure 4:**
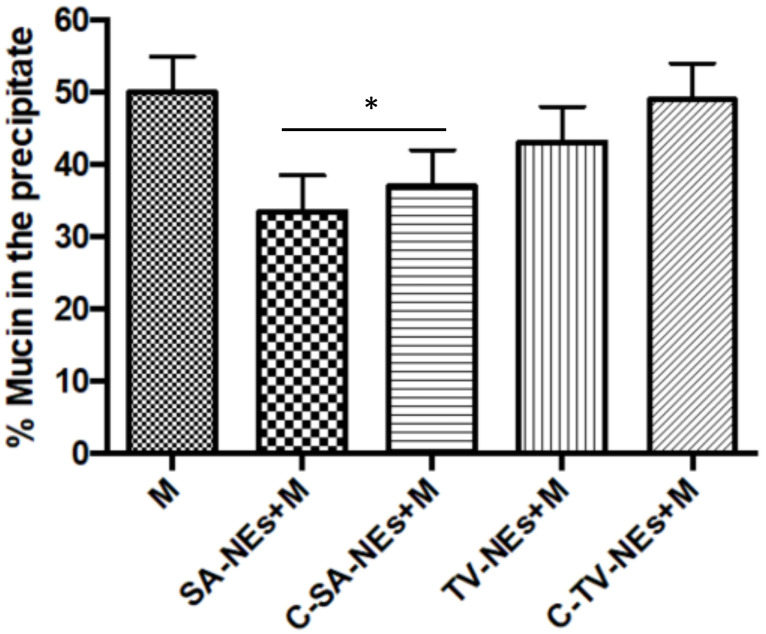
Mucoadhesive efficiency of nanoemulsions: Mucin interaction evaluating the percent of precipitated mucin (* = *p* < 0.05 SA samples when compared with mucin).

The interaction between mucin and NEs was evaluated by measuring the UV spectra of the supernatant after incubation and centrifugation. The percentage of mucin in the precipitate was monitored measuring the difference between the total amount of mucin and the free mucin present in the supernatant (Figure 4). In the presence of only mucin in the suspension, a precipitation of 50% was observed after centrifugation, while when coated and uncoated NEs were included in the suspension, a decrease in the precipitated percentage was noted, likely as a consequence of a mechanical interaction between NEs and mucin. Due to their low density, NEs remain in the supernatant together with mucin that remains adherent to the structure [42].

### 3.6. In Vitro Release Study

**Figure 5:**
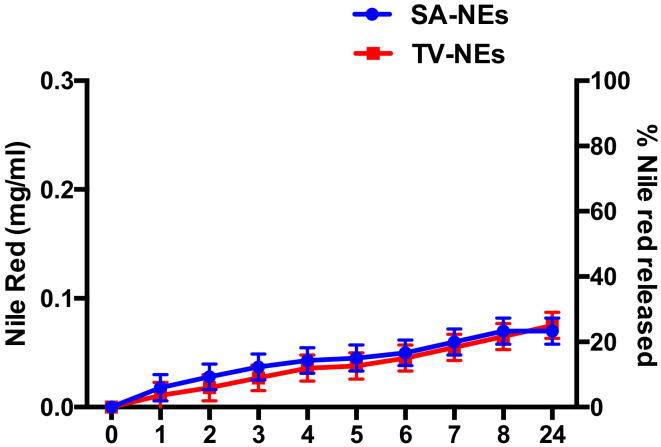
Profile of Nile Red released by NEs expressed as a concentration of Nile Red in the external medium and as a % of Nile Red released.

Release studies were carried out as above described. Release profiles of NR by uncoated NEs are shown in Figure 5. Release studies, antimicrobial activity, and time-kill studies were carried out only with uncoated nanoemulsions, because probably, the coating should be retained on the olfactory epithelium, and only NEs or free drug should be able to cross the nasal mucosa. From the obtained results, it is possible to affirm that a certain amount of NR present in the formulation was released in the acceptor compartment. The amount of NR loaded in NEs is low enough to not vary nanodroplet features and its entrapment efficiency is around 100%. The low aqueous solubility and loaded amount are responsible of the low release in the external medium [44].

### 3.7. Antimicrobial Activity of NEs

The TV-NE and SA-NE were tested for their antimicrobial efficacy against all selected strains and evaluated by means of MIC (data not shown) and MBC. For comparison purposes the untreated essential oils, TVEO and SAEO, and the most abundant chemical components thymol and eugenol were also investigated.

NEs showed potent activities (expressed in % *v/v*) against all the tested bacterial strains (Table 7) with MBC values ranging from 0.125 to 0.03% *v/v*, although slightly decreased with respect to the untreated EOs that showed MIC and MBC values ranging from 0.07 to less than 0.03% *v/v*. In particular, MIC/MBC values for CR-Ab and CR-Kp for TV-NEs and SA-NEs had the same value, corresponding to 0.03% *v/v*. Interestingly, thymol and eugenol showed unpredictable behavior. In particular thymol, the TVEO major component (>80%), showed average MIC and MBC values of 0.031% *v/v* and 0.039% *v/v* slightly higher than the approximated corresponding average values for TVEO, indicating that some other chemical components could act synergistically in the whole EO and importantly the activity is somehow retained in the TV-NE. A similar scenario was observed for eugenol, the most abundant SAEO component (>44%), but due to the less percentage the synergistic effect was less marked as eugenol alone was usually found more potent than SAEO and SA-NE (Table 7).

The antimicrobial activity was also checked in a standard DD assay confirming the biological activity profiles of TVES and SAEO observed in the microdilution experiments (Table 8).

### 3.8. Time-Kill Studies of NEs

**Figure 6:**
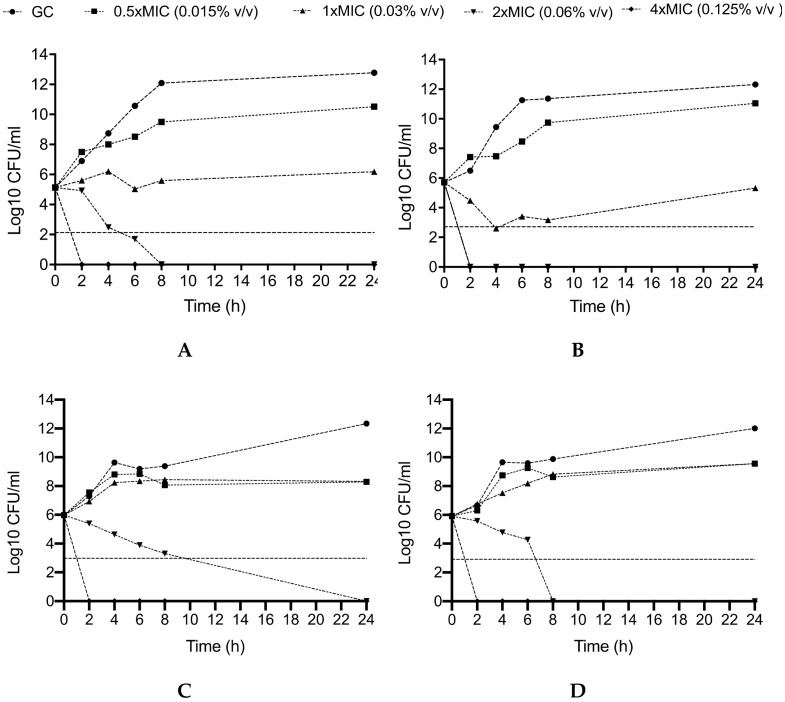
Activity SA-NEs and TV-NEs against CR-Ab (**A,B**) and CR-Kp (**C,D**) at different concentrations throughout the killing study. GC: Growth Control. The horizontal dashed line represents bactericidal activity. CR-Ab: Carbapenem-resistant Acinetobacter baumannii; CR-Kp: Carbapenem-resistant Klebsiella pneumoniae. The MIC values of CR-Ab and CR-Kp for SA-NEs and TV-NEs were 0.03% *v/v* and 0.03% *v/v*.

In agreement with the MBC data, killing studies of SA- and TV-NEs showed a remarkable anti-bacterial activity against CR-Ab and CR-Kp, with a concentration-dependent effect (Figure 6). Against CR-Ab (Panel A, B), SA-NEs was bactericidal after 2 and 6 h at the concentrations 0.125% and 0.06% *v/v*, respectively, with an absence of bacterial growth, whereas TV-NEs was rapidly bactericidal after 2 h of incubation at the concentration of 0.06% *v/v*.

On the other hand, against CR-Kp (Panel C, D) the bactericidal activity of SA-NEs and TV-NEs at the concentration of 0.06% *v/v* was slightly slower (24 and 8 h, respectively), whereas the concentration 0.125% *v/v* of both SA- and TV-NEs maintained an early and durable bactericidal activity, showing an absence of bacterial growth after 2 h.

## 4. Conclusions

The two analyzed EOs show different chemical compositions, responsible for different antimicrobial activities of the untreated EOs and of the SA- and TV-NEs. Furthermore, the appropriate mixing of each EO with surfactants led to the formation of O/W nanoemulsions with a droplet hydrodynamic diameter in the nanometric range and negative ζ-potential able to assure a high storage stability of the proposed nanoformulations. Chitosan coated nanoemulsions were also prepared and characterized. Chitosan-NEs show good mucoadhesive properties and pH values compatible with nasal administration. As highlighted by the in vitro experiments, chitosan NEs may represent a promising solution against bacterial strains of clinical concern such as methicillin-susceptible and methicillin-resistant *Staphylococcus aureus*, *Escherichia coli*, and multi-drug resistant Gram-negative bacteria including CR-Ab and CR-Kp, commonly encountered as the causative pathogens of nosocomial (i.e., post-surgical) meningitis. In fact, the presence of BBB, although altered in the presence of inflammation/infection, is the major limitation for drugs and antibiotics to reach the subarachnoid space. An additional way of local antimicrobial administration is represented by the intrathecal route, which is however limited by possible toxic effects and the need of an intraventricular catheter. Therefore, finding alternative and effective ways of drug administration seems to be crucial.

Data in the literature have been mainly focused on animal infection models of nose-to-brain delivery of ketoconazole in cryptococcal meningoencephalitis [7] or on adjunctive and long-acting nanoformulated antiretroviral therapies for HIV-associated neurocognitive disorders [9,45,46], whereas data regarding bacterial infections are scarce [7]. NEs described in this study appear suitable to be administered intranasally and indicated potential benefits over other types of delivery systems. Titled NEs could be used to develop an alternative and efficient therapy for the treatment of brain infections and in particular on the management of meningitis and encephalitis by means of a N2B administration as a possible add-on strategy in combination with the traditional intravenous high dose of appropriate antimicrobials.

A wide range of oils, essential oils, and emulsifiers, can be chosen, trying to couple the activity of the oil with the loaded drug in order to achieve a potentiated synergic effect. Furthermore, the feasibility of NE preparation makes them an optimum choice as their production can be scaled up using specific processing operations that are already commonly used in the pharmaceutical and food industry, such as high-pressure homogenization, microfluidization, and sonication.

## Figures and Tables

**Table 1:** Nanoemulsions (NEs) composition.

Sample	Span20 (mg/mL)	SAEO (mg/mL)	TVEO (mg/mL)	Chitosan (mg/mL)
SA-NEs	5.2	5.2	-	-
C-SA-NEs				0.06
TV-NEs		-	5.2	-
C-TV-NEs				0.06

**Table 2:** Chemical composition (%) of *Syzygium aromaticum* essential oil (SAEO) (A1) and *Thymus vulgaris* essential oil (TVEO) (A2).

N°	Component	LRI ^1^	LRI ^2^	A1%	A2%
1	α-pinene	1020	1021	-	1.3 ± 0.64
2	camphene	1063	1065	-	1.0 ± 0.11
3	β-pinene	1090	1099	-	0.3 ± 0.09
4	β-myrcene	1162	1157	-	1.1 ± 0.05
5	α-terpinene	1180	1186	-	0.6 ± 0.04
6	limonene	1190	1198	-	0.5 ± 0.02
7	eucalyptol	1200	1209	-	1.2 ± 0.08
8	γ-terpinene	1241	1244	-	5.2 ± 0.19
9	o-cymene	1280	1287	-	18.2 ± 0.31
10	1-octen-3-ol	1465	1458	-	0.5 ± 0.11
11	camphor	1512	1507	-	2.4 ± 0.02
12	linalool	1542	1537	-	7.5 ± 0.30
13	methyl thymyl ether	1550	1555	-	0.4 ± 0.03
14	terpinen-4-ol	1610	1603		3.0 ± 0.11
15	β-caryophyllene	1622	1619	8.7 ± 0.20	2.4 ± 0.03
16	cis-beta-terpineol	1650	1644 *	-	0.2 ± 0.02
17	humulene	1675	1668	1.0 ± 0.03	-
18	α-terpineol	1698	1690 *	-	0.2 ± 0.06
19	endo-borneol	1720	1717	-	2.2 ± 0.02
20	methylsalicylate	1768	1763	0.2 ± 0.03	-
21	caryophyllene oxide	1896	1892	-	0.7 ± 0.03
22	thymol	2158	2154	-	44.4 ± 0.17
23	eugenol	2180	2172	80.1 ± 0.46	-
24	carvacrol	2230	2222	-	6.6 ± 0.04
25	eugenol acetate	2281	2277 *	9.9 ± 0.79	-
	Sum			99.9	99.9

^1^ Linear retention indices (LRI) measured on a polar column; ^2^ LRI from the literature; * Normal alkane RI.

**Table 3:** DLS analyses on SA- and TV-NEs in the presence and absence of chitosan. Values represent the mean ± standard deviation of n = 3 NE sample measurements.

Sample	Hydrodynamic Diameter (nm) ± SD	ζ-Potential (mV) ± SD	PDI ± SD
SA-NEs	106.0 ± 0.2	−45.3 ± 0.5	0.21 ± 0.04
C-SA-NEs	238.0 ± 6.2	+38.1 ± 4.9	0.25 ± 0.07
TV-NEs	110.0 ± 2.9	−48.3 ± 0.4	0.22 ± 0.02
C-TV-NEs	232.0 ± 8.2	+40.3 ± 1.2	0.24 ± 0.08

**Table 4:** NE oil phase characterization by fluorimetric assays utilizing two probes: Pyrene for microviscosity and polarity assessment and DPH for fluidity evaluation.

Sample	Polarity (I_1_/I_3_)	Microviscosity (I_E_/I_3_)	Fluidity (Anisotropy)
SA-NEs	0.92	1.72	0.24
C-SA-NEs	0.83	1.77	0.21
TV-NEs	0.88	2.04	0.26
C-TV-NEs	0.79	2.05	0.34

**Table 5:** Characteristics of nanoemulsions before and after contact with the mucin dispersion.

Sample	Hydrodynamic Diameter (nm)	ζ-Potential (mV) ± SD	PDI ± SD	pH	Turbidity (AU)
SA-NEs	106.0 ± 0.2	−45.3 ± 0.5	0.21 ± 0.04	6.2	283.4
SA-NEs + M	167.2 ± 2.3	−34.1 ± 1.2	0.23 ± 0.09	6.4	321.3
TV-NEs	110.0 ± 2.9	−48.3 ± 0.4	0.22 ± 0.02	6.4	318.8
TV-NEs + M	146.1 ± 3.4	−24.8 ± 0.7	0.26 ± 0.09	6.3	344.0
M	1623.0 ± 57.0	−15.6 ± 0.4	0.4 ± 0.09	6.1	346.3

**Table 6:** Characteristics of chitosan coated nanoemulsions before and after contact with the mucin dispersion.

Sample	Hydrodynamic Diameter (nm)	ζ-Potential (mV) ± SD	PDI ± SD	pH	Turbidity (AU)
C-SA-NEs	238.0 ± 6.2	+38.1 ± 4.9	0.25 ± 0.07	6.3	101.5
C-SA-NEs + M	252.7 ± 8.3	−35.5 ± 0.9	0.28 ± 0.07	6.2	119.7
C-TV-NEs	232.0 ± 8.2	+40.3 ± 1.2	0.24 ± 0.08	6.3	99.1
C-TV-NEs + M	234.9 ± 9.1	−35.7 ± 2.3	0.29 ± 0.09	6.2	150.6
M	1623.0 ± 57.0	−15.6 ± 0.4	0.4 ± 0.10	6.1	346.3

**Table 7:** Antimicrobial activities MIC/MBC (% *v/v*) of either TVEO or SAEO containing NEs in comparison with free EOs and their major components thymol and eugenol. Vancomycin and meropenem were used as positive controls for Gram-positive and Gram-negative bacteria, respectively.

Strains	MSSA		MRSA		CR-Ab		CR-Kp		CR-Pa		*E. coli*	
	MIC	MBC	MIC	MBC	MIC	MBC	MIC	MBC	MIC	MBC	MIC	MBC
TV-NEs	0.125	0.125	>0.125	>0.125	0.03	0.03	0.03	0.03	0.125	0.125	>0.125	>0.125
TVEO	<0.03	<0.03	<0.03	<0.03	<0.03	<0.03	<0.03	<0.03	<0.03	<0.03	<0.03	<0.03
Thymol *	0.031	0.062	<0.015	0.031	<0.015	<0.015	0.062	0.062	0.031	0.031	0.031	0.031
SA-NEs	0.125	0.125	>0.125	>0.125	0.03	<0.03	0.03	0.03	0.125	0.125	0.125	0.125
SAEO	<0.03	0.07	<0.03	0.07	<0.03	0.07	<0.03	0.07	0.07	0.07	<0.03	0.07
Eugenol *	0.007	<0.003	0.007	0.007	<0.003	<0.003	0.007	0.007	0.056	0.056	0.028	0.028
VAN *^,^**	0.00004	0.00007	0.00007	0.00007	NA	NA	NA	NA	NA	NA	NA	NA
MEM *^,^**	NA	NA	NA	NA	0.005	0.009	0.018	0.037	0.0006	0.001	0.000004	0.000004

MSSA: Methicillin-sensitive Staphylococcus aureus; MRSA: Methicillin-resistant Staphylococcus aureus; CR-Ab: Carbapenem-resistant Acinetobacter baumannii; CR-Kp: Carbapenem-resistant Klebsiella pneumoniae; CR-Pa: Carbapenem-resistant Pseudomonas aeruginosa. MIC: Minimal Inhibitory Concentration; MBC: Minimal Bactericidal Concentration; VAN: vancomycin; MEM: meropenem; NA: Not Active; * For pure compounds and references the concentration was expressed as mg/mL; ** data taken from [25].

**Table 8:** Microbiological evaluation by disk diffusion (DD in mm) for TVEO and SAEO.

Strains	TVEO	SAEO
MSSA	29	6
MRSA	29	8
CR-Ab	28	12
CR-Kp	26	10
CR-Pa	0	0
*E. coli*	24	7

MSSA: Methicillin-sensitive Staphylococcus aureus; MRSA: Methicillin-resistant Staphylococcus aureus; CR-Ab: Carbapenem-resistant Acinetobacter baumannii; CR-Kp: Carbapenem-resistant Klebsiella pneumoniae; CR-Pa: Carbapenem-resistant Pseudomonas aeruginosa.
